# Navigating the Intersection of Monkeypox and Pregnancy: A Call for Vigilance and Research

**DOI:** 10.7759/cureus.68615

**Published:** 2024-09-04

**Authors:** Athina Diamanti, Chrysoula Taskou, Antigoni Sarantaki

**Affiliations:** 1 Department of Midwifery, University of West Attica, Athens, GRC; 2 Department of Midwifery, General Hospital Alexandra, Athens, GRC

**Keywords:** vertical transmission, fetal health, maternal health, pregnancy, monkeypox

## Abstract

The recent global spread of monkeypox has raised significant concerns, particularly regarding its impact on vulnerable populations such as pregnant individuals. While limited data suggest possible adverse outcomes, including vertical transmission and fetal demise, there remains a critical need for comprehensive research to inform clinical management and public health strategies. The lack of specific guidelines and tailored public health messaging for pregnant individuals underscores the urgency for focused attention in this area. Addressing these gaps is essential to ensuring the health and safety of both mothers and their unborn children during monkeypox outbreaks.

## Editorial

Monkeypox, a viral zoonosis endemic to Central and West Africa, has recently emerged as a global health concern, particularly in light of the 2022 outbreak that extended far beyond its usual geographic confines. In 2022, the global monkeypox outbreak resulted in more than 83,000 confirmed cases across 110 countries. While the virus is known to cause a disease similar to smallpox, with symptoms including fever, rash, and lymphadenopathy, its implications for specific populations, such as pregnant individuals, remain inadequately explored [1].

Pregnancy is a state of altered immunity, characterized by a complex balance between immune tolerance to the fetus and the capacity to combat infections. This immunological shift makes pregnant individuals particularly vulnerable to certain infectious diseases, as evidenced by the heightened risks associated with influenza, Zika virus, and COVID-19 during pregnancy. In the context of monkeypox, the potential risks to both the pregnant individual and the developing fetus are concerning yet poorly understood. The potential risks of monkeypox to both pregnant individuals and their developing fetuses are concerning due to the possibility of vertical transmission, which can lead to severe outcomes like fetal demise or congenital infection. Pregnancy involves significant immunological changes that may increase susceptibility to infections like monkeypox, potentially worsening the disease's impact [2].

Emerging data from the recent outbreak and previous case reports suggest that monkeypox can indeed pose specific risks during pregnancy, including vertical transmission of the virus from the mother to the fetus, which can result in severe outcomes such as fetal demise, congenital infection, and premature delivery. These risks are especially concerning given the altered immune state during pregnancy, which may heighten the susceptibility to infections like monkeypox and potentially exacerbate the severity of the disease. These reports, though limited, underscore the need for heightened vigilance and more extensive research to delineate the full spectrum of risks associated with monkeypox in pregnancy [3]. One of the critical challenges in addressing this issue is the paucity of data. The limited number of cases involving pregnant individuals hampers our understanding of how monkeypox affects pregnancy and what measures can be taken to mitigate these risks.

Additionally, the lack of specific guidelines for managing monkeypox in pregnant patients further complicates the situation. Current treatment protocols for monkeypox do not account for the unique needs of pregnant individuals, particularly concerning the safety and efficacy of antiviral therapies and the use of immunoglobulins during pregnancy. Current treatment protocols for monkeypox primarily involve the use of antiviral therapies, such as tecovirimat, and supportive care measures. These treatments aim to reduce the severity and duration of symptoms. Additionally, vaccinia immunoglobulin is sometimes considered for severe cases or for those with weakened immune systems. However, these protocols do not specifically address the unique needs of pregnant individuals. The safety and efficacy of these antiviral treatments and immunoglobulins during pregnancy are not well established, which raises concerns. Pregnant individuals may experience altered drug metabolism and immune responses, which could affect how these treatments work and their potential risks to the fetus. Without targeted research and guidelines, healthcare providers face significant uncertainty in managing monkeypox in pregnant patients, leading to suboptimal care and potential risks for both the mother and the developing fetus​ [4].

In the absence of robust data, healthcare providers are left to navigate these cases with considerable uncertainty. This uncertainty extends to decisions regarding the timing and mode of delivery, the management of potential complications, and the protection of newborns from infection in the postpartum period. The lack of clear guidelines underscores the need for urgent research to inform clinical practice and ensure that pregnant individuals receive the best possible care [5]. Moreover, public health messaging must be tailored to address the concerns of pregnant individuals. The current communication strategies around monkeypox often do not adequately address the specific risks and precautions for this population. Pregnant individuals need clear, evidence-based guidance on how to protect themselves from infection, the signs and symptoms to watch for, and what to do if they suspect they have been exposed to the virus. Public health agencies must prioritize the development of such guidance as part of their broader monkeypox response efforts [5].

Currently, there are significant gaps in understanding the precise mechanisms of vertical transmission of monkeypox during pregnancy, the specific gestational stages that might be most vulnerable, and the long-term outcomes for infants born to mothers who contracted the virus during pregnancy. Additionally, there is a need to explore the safety and efficacy of existing antiviral therapies and immunoglobulins, specifically in pregnant populations. Research could also focus on developing new therapeutic interventions or preventive measures tailored to the unique immunological environment of pregnancy. Addressing these gaps through rigorous studies would be essential for informing clinical guidelines that can better protect both pregnant individuals and their babies from the risks associated with monkeypox.

In conclusion, the intersection of monkeypox and pregnancy represents a critical area of concern that demands immediate attention from the global health community. The potential risks to maternal and fetal health necessitate a proactive approach, combining rigorous research with the development of targeted clinical guidelines and public health messaging. As we continue to confront the challenges posed by emerging infectious diseases, it is imperative that we do not overlook vulnerable populations such as pregnant individuals. Only through concerted effort and collaboration among global health organizations, researchers, and healthcare providers can we ensure that pregnant individuals are fully protected from the risks associated with monkeypox, ultimately leading to healthier outcomes for both mothers and their babies. This collective approach is essential for developing targeted research, clinical guidelines, and public health messaging that address the unique vulnerabilities of pregnancy during monkeypox outbreaks, thereby safeguarding the health of future generations.
